# Bone resorption predicts for skeletal complications in metastatic bone disease

**DOI:** 10.1038/sj.bjc.6601437

**Published:** 2003-11-25

**Authors:** J E Brown, C S Thomson, S P Ellis, S A Gutcher, O P Purohit, R E Coleman

**Affiliations:** 1 Academic Unit of Clinical Oncology, Cancer Research Centre, Weston Park Hospital, Sheffield S10 2SJ, UK; 2 Trent Cancer Registry, Weston Park Hospital, Sheffield S10 2SJ, UK

**Keywords:** metastatic bone disease, bone markers, N-telopeptide, Ntx, skeletal-related events

## Abstract

Relationships between the rate of bone resorption (measured by urinary N-telopeptide (Ntx) excretion) and a range of skeletal complications have been evaluated in patients with metastatic bone disease. A total of 121 patients had monthly measurements of Ntx during treatment with bisphosphonates. All skeletal-related events, plus hospital admissions for bone pain and death during the period of observation, were recorded. Data were available for 121 patients over the first 3-month period of monitoring (0–3 months) and 95 patients over the second 3-month period (4–6 months). N-telopeptide levels were correlated with the number of skeletal-related events and/or death (*r*=0.62, *P*<0.001 for 0–3 months and *r*=0.46, *P*<0.001 for 4–6 months, respectively). Patients with baseline Ntx values ⩾100 nmol mmol^−1^ creatinine (representing clearly accelerated bone resorption) were 19.48 times (95% CI 7.55, 50.22) more likely to experience a skeletal-related event/death during the first 3 months than those with Ntx <100 (*P*<0.001). In a multivariate logistic regression model, Ntx was highly predictive for events/death. This study is the first to indicate a strong correlation between the rate of bone resorption and the frequency of skeletal complications in metastatic bone disease. N-telopeptide appears useful in the prediction of patients most likely to experience skeletal complications and thus benefit from bisphosphonate treatment.

Metastatic bone disease presents a major challenge in the management of several common cancers. Tumours of the breast and prostate are particularly likely to metastasise to bone, with up to 70% of patients dying with advanced metastatic disease showing evidence of skeletal involvement at post mortem (Rubens, 2000). Skeletal complications include hypercalcaemia, bone pain requiring radiotherapy, pathological fracture and spinal cord or nerve root compression. Additionally, a proportion of patients will die as a direct consequence of their skeletal disease. Owing to the prolonged course of metastatic bone disease (Coleman and Rubens, 1987), the frequency of skeletal events in relation to average patient survival assumes particular importance. Without bisphosphonate therapy, a major skeletal event occurs on average once every 3–4 months (Hortobagyi *et al*, 1996; Theriault *et al*, 1999). This can seriously diminish a patient's quality of life, as well as having huge resource implications for health services (Richards *et al*, 1993).

Metastatic bone disease is associated with a disruption of the normal coupling between bone formation and resorption, typically resulting in net osteolysis leading to loss of structural integrity and subsequent skeletal events. Bone-targeted drug therapy, principally using the bisphosphonates, has been aimed at disruption of this osteoclast mediated bone resorption. Trials carried out over the last decade have provided overwhelming evidence supporting the use of bisphosphonates in reducing the morbidity of metastatic bone disease from across a range of tumour types (Hortobagyi *et al*, 1996; Berenson *et al*, 2001; Rosen 2002; Saad *et al*, 2002). However, despite the overall clinical benefits of bisphosphonates, it is currently impossible to predict whether an individual patient will benefit from a bisphosphonate.

Type I collagen is the predominant protein in bone and its breakdown products are being increasingly investigated as markers of bone resorption. A number of studies have shown that collagen breakdown markers are correlated with the presence of metastatic disease. N-telopeptide (Ntx) is a peptide fragment of the N-terminus of type I collagen, and is relatively easy to measure. It appears to be one of the most potentially useful of these markers in metastatic bone disease (Lipton *et al*, 2001). In recent years, clinical studies have demonstrated a correlation between bone resorption markers, especially Ntx, and the presence and extent of metastases, prognosis and response to treatment (Lipton *et al*, 2001). A study by Vinholes *et al* (1996) showed a significant correlation between metastatic bone pain and bone resorption. Several subsequent studies in both breast and prostate cancer patients have confirmed this initial finding (Jagdev *et al*, 2001). Both oral and intravenous bisphosphonate therapy have been shown to reduce Ntx levels and, importantly, only those patients who showed normalisation of bone resorption following bisphosphonate treatment experienced clinical benefit in terms of an improvement in pain, analgesic use and morbidity (Vinholes *et al*, 1997).

In osteoporosis, a clear relationship has been established between levels of bone markers such as Ntx and the occurrence of skeletal complications, whether or not a patient has received bone-specific therapy. However, with few exceptions (Lipton *et al*, 1998; Menssen *et al*, 2002), little work has been carried out on the possible correlation between bone markers and the occurrence of skeletal events in metastatic bone disease. If such a link can be firmly established, then it may be appropriate to direct the management of patients with metastatic bone disease to maintain or lower an individual's bone marker values into the normal range. This study seeks to determine if there is such a relationship between Ntx levels and subsequent skeletal complications in patients with metastatic bone disease.

## METHODS

### Patients

A total of 121 unselected, consecutive patients with histologically proven malignancy and bone metastases, confirmed by appropriate imaging (plain X-ray, CT scan or MRI), were prospectively studied in the Cancer Research Centre in Sheffield, a regional referral centre for patients with bone metastases. All patients were over 18 years of age and had signed informed consent according to local guidelines with the approval of the appropriate Ethics Committee. Patients with lytic, sclerotic and mixed lesions as defined radiologically were included. Patient characteristics are shown in Table 1

**Table 1:** Baseline patient characteristics

**Tumour type**	**Number of patients**	**Median age in years**	**Median time from cancer diagnosis to diagnosis of bone metastasis in months (range)**	**Time from diagnosis of bone metastasis to baseline Ntx in months (range)**	**Number of patients with metastasis at other sites at baseline**
Breast	91	55.0	37.0 (0.0, 187.1)	9.9 (0.0, 65.1)	24
Prostate	26	69.5	3.5 (0.0, 96.0)^a^	21.5 (4.0, 101.0)	3
Other^b^	4	48.0	8.5 (1.0, 206.0)	12.5 (1.0, 19.3)	2
Total	121	56.0	30.5 (0.0, 206.0)	10.4 (0.0, 101.0)	29

a This apparently low value is because 50% of the prostate cancer patients had bone metastases present at the time of diagnosis of their primary cancer.

b Includes 1 lung, 1 renal, 1 thyroid and 1 Sertoli cell tumour.

.

Measurements of the bone resorption marker Ntx were carried out in all 121 patients. The date of the first Ntx measurement was recorded for each patient as the baseline value (month zero). Further measurements were taken at approximately monthly intervals. All 121 patients had Ntx measurements available over at least 3 months or until death if this occurred first. In all, 95 patients were evaluable for at least 6 months. Where possible, patients were followed up for a maximum of 24 months, but the number of patients available to the study decreased considerably over the later months due to progression of disease or return to a routine clinic outside Sheffield.

In all, 29 patients also had extra-skeletal metastases at baseline, most commonly affecting the liver, but including lung and lymph node involvement. All patients in the study received bisphosphonate therapy and 93 patients had not received any such treatment before the first Ntx measurement. Most patients (75) were treated with oral clodronate (1600 mg); other treatments included IV pamidronate, 90 mg (29 patients), IV clodronate, 1500 mg (eight patients) and IV zoledronic acid, 4 mg (nine patients). Patients were treated with endocrine or chemotherapy treatments as clinically indicated.

### Ntx measurement

The bone resorption was assessed approximately monthly by measurement of urinary crosslinks in an early morning, second voided urine sample, collected on the day of outpatient attendance and stored at −20°C for later analysis. These measurements were made using a chemiluminescence assay for the Ntx using a Vitros ECI analyser (Hanson *et al*, 1992).

The coefficient of variation of the test varies from 6.8 to 14.9% across the range of 96–1928 nmol bone collagen equivalents. The Ntx normal ranges were based on normal male (71 subjects) and female controls (58 premenopausal and 92 postmenopausal subjects) in the regional population, not known to have bone disease or to be taking drugs known to affect bone metabolism. None had had recent fractures. The resultant Ntx normal range used in our centre was: male 16–107 nmol mmol^−1^ creatinine and female 10–87 nmol mmol^−1^ creatinine for premenopausal women and 32–124 nmol mmol^−1^ creatinine for postmenopausal women. A level of 100 nmol mmol^−1^ creatinine was selected as a reasonable simple approximation of the upper limit of normal across the study population.

### Skeletal complications

For all patients, any skeletal complications occurring after the initial Ntx measurement were recorded. Skeletal complications were defined as radiotherapy to bone, hypercalcaemia (corrected serum calcium concentration ⩾2.7 mmol l^−1^), spinal cord or nerve root compression, symptomatic radiographically confirmed pathological fractures, orthopaedic surgery to bone, hospital admissions for control of bone pain and/or death due to metastatic bone disease. It was considered important to include death as a skeletal complication, as patients with the worst prognosis were more likely to die early either before or as a consequence of a particularly catastrophic skeletal event. Routine plain radiographs were not obtained, therefore excluding the detection of asymptomatic fractures. As skeletal-related events may be inter-related (e.g. pathological fracture followed by surgery followed by radiotherapy), the primary, more conservative analysis took into account only the first event in any 21-day period. However, the total number of events, not taking account of the 21-day window, was also recorded.

### Statistical methods

Spearman's rank correlation coefficients were obtained to assess whether there were relationships between the baseline Ntx, or the mean of the Ntx values between 0 and 3 months, and the numbers of skeletal complications during the subsequent 3 months. These were also obtained for Ntx at 3 months, or the mean of the Ntx values between 4 and 6 months, and the numbers of skeletal complications during months 4–6. *χ*^2^ tests of association were performed on these various Ntx measures, grouped into four categories, and whether or not a patient had one or more skeletal complication.

Univariate and multivariate logistic regression models (Armitage and Berry, 1994) were fitted to determine as to whether Ntx measurements could predict a skeletal event and/or death over a 3-month time period. A 3-month time period was considered as the maximum interval that one might expect a single biochemical measurement to reflect accurately the underlying metabolic bone state. In the multivariate analyses, the other factors included in the models were age, sex and cancer type. Adjusted odds ratios were presented for each level of Ntx measurements compared with a reference category. No adjustment was made for multiple testing.

## RESULTS

### Distribution of skeletal complications and dependence upon Ntx values

The distribution of the type of skeletal complication over the 0–6-month time period is shown in Table 2

**Table 2:** Distribution among the various types of skeletal complication during the 0–6 month period

**Skeletal event and/or death**	**Number of events**	**Median (and range) of Ntx value**
Fracture	19	231 (47–698)
Cord compression	4	232 (140–1762)
Hypercalcaemia	7	273 (146–683)
Radiotherapy	35	164 (26–720)
Admission for pain control	15	193 (45–1412)
Death	21	199 (30–683)

**Figure 1:**
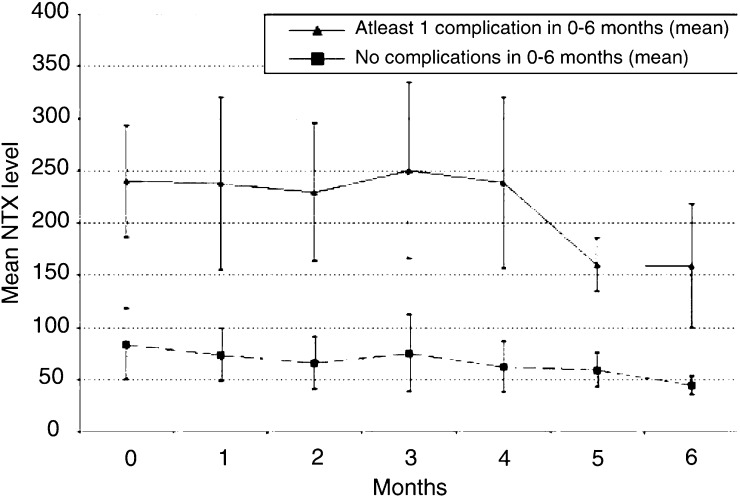
Mean Ntx values *vs* time (bars show 95% confidence intervals) for patients with (–▴–) or without (**–▪–**) a skeletal complication over the period 0–6 months.

The table shows the median of the Ntx value that was recorded in the month prior to the event, as well as minimum and maximum values of Ntx for each type of event. Note that these counts are not mutually exclusive as several patients had more than one type of event during the time period 0–6 months. The analysis includes the 111 patients who remained on study or died during the first 6 months.

in relation to the Ntx value measured in the month before the event occurred. The data are not mutually exclusive, since several patients had more than one type of event and eight patients had more than one episode of the same type of event. Consistent with other studies (LoRusso, 2001), the most frequent skeletal event was radiotherapy to bone, followed by fracture or orthopaedic surgery to bone, with spinal cord or nerve root compression and hypercalcaemia relatively less frequent. For every type of skeletal complication, the median Ntx value was substantially above the normal range, for the less common but extreme events of spinal cord compression and hypercalcaemia, all Ntx values were above the normal range. Figure 1

shows the mean (and 95% confidence intervals) for Ntx values at monthly intervals over 6 months for those patients with and without one or more skeletal complication. A clear distinction in Ntx values for the two groups is seen.

Table 3

**Table 3:** Distribution of skeletal complications excluding and including death during 0–3 months and 4–6 months

	**0–3 months**		**4–6 months**	
**Number of events**	**Number of patients excluding death (including death)**	**%**	**Number of patients excluding death (including death)**	**%**
Zero	75 (72)	62.0 (59.5)	73 (63)	76.8 (66.3)
One	31 (31)	25.6 (25.6)	20 (26)	21.1 (27.4)
Two	10 (12)	8.3 (9.9)	2 (6)	2.1 (6.3)
Three	5 (6)	4.1 (5.0)	0 (0)	0 (0)
Total number of patients	121 (121)	100 (100)	95 (95)	100 (100)

Total number of complications: 66 (73) (0–3 months), 24 (38) (4–6 months).

shows the distribution of the numbers of skeletal complications occurring during the first (0–3 months) and second (4–6 month) 3-month time periods. In all, 73 and 38 events were recorded in the first and second 3-month time periods, respectively. Overall, there was a total of 111 complications in the 121 patients over the 6 months. In all, 21 patients (17%) died during the first 6 months of observation, four before experiencing a specific skeletal event.

Relaxation of the rule ignoring additional complications occurring within a 21-day window produced an additional 13 skeletal events. Almost all of these occurred in the first 3 months (three orthopaedic surgery following fractures, six radiotherapy after spinal cord compression and four admissions for pain control after fractures). Since this study was primarily concerned with relationships to one or more events, the remainder of the analyses refer to the data with the 21-day window applied.

### Analysis of 0–3 month data

Figure 2A

**Figure 2:**
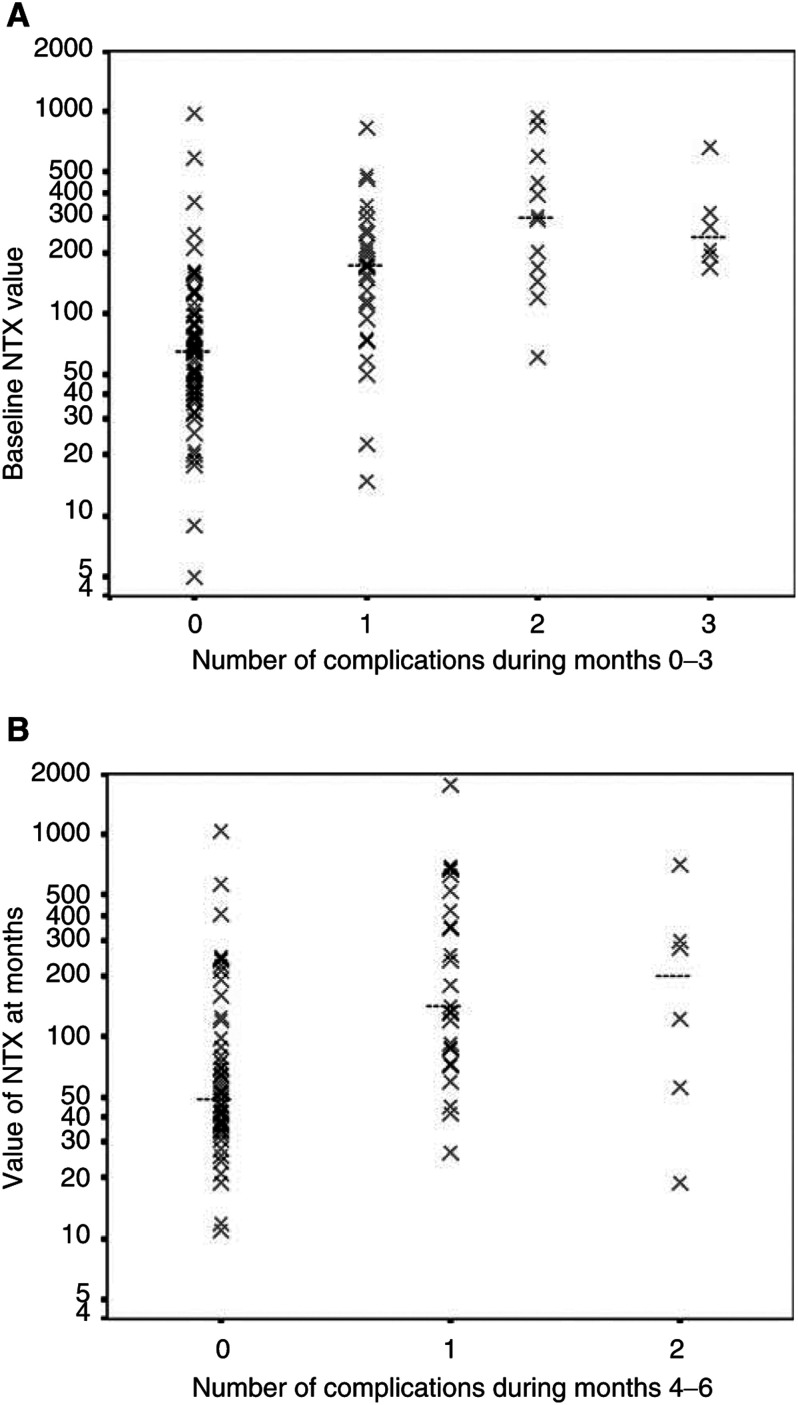
Scatter plots of baseline Ntx (log scale) against the number of skeletal complications during (**A**) 0–3 months and (**B**) 4–6 months. Median values of Ntx in nmol mmol^−1^ creatinine were 65.5 (no skeletal complication), 171 (one skeletal complication), 298.5 (two skeletal complications) and 240.5 (three skeletal complications) in (**A**) and 49.0 (no skeletal complication), 142 (one skeletal complication) and 197.5 (two skeletal complications) in (**B**).

presents a scatter plot of Ntx at baseline (using a logarithmic scale) against the number of skeletal complications during 0–3 months. While there is evidently a wide scatter of Ntx values, nevertheless these data show a clear relationship between the number of complications and the Ntx value. The Spearman rank correlation coefficient (*r*) is 0.62 (*P*<0.001). A similar plot for the 0–3-month period using the mean Ntx during the first 3 months (derived from the four measurements at baseline and 1, 2 and 3 months) instead of Ntx at baseline was also strongly correlated with the number of skeletal complications (*r*=0.66, *P*<0.001). However, the single baseline Ntx is more clinically useful as it enables prediction for events over the subsequent three months.

Interpretation of the predictive value of Ntx as a continuous variable is difficult, and therefore the Ntx values were grouped. Four groups of Ntx values were defined: 0–50 nmol mol^−1^ creatinine, corresponding approximately to the normal male and premenopausal female levels; 50–100 nmol mol^−1^ creatinine, corresponding closely to the upper normal range for postmenopausal women; 100–200 nmol mol^−1^ creatinine, defining a group with moderately accelerated bone resorption; and >200 nmol mol^−1^ creatinine, representing very rapid bone resorption. Table 4A

**Table 4:** Distribution of skeletal complications and unadjusted and adjusted odds ratios according to (A) baseline Ntx range for the 0–3 month time period and (B) the 3-month value for the 4–6-month time period

**(A) baseline Ntx range for the 0–3 month time period**							
**Baseline Ntx/Ntx value at 3 months (nmol/mmol creatinine)**	**Number of patients**	**Number of patients having any skeletal complication**	**% patients having any skeletal complication**	**Unadjusted odds ratio**	**95% confidence interval**	**Adjusted odds ratioa**	**95% confidence interval**
0–50	31	3	9.7	1	^*^	1	^*^
>50–100	34	5	14.7	1.61	0.35, 7.38	1.51	0.31, 7.45
>100–200	26	16	61.5	14.93	3.58, 62.34	14.24	3.37, 60.17
>200	30	25	83.3	46.67	10.11, 215.42	42.17	9.01, 197.45
Total	121	49	40.5	^*^	^*^	^*^	^*^
							
3-month value for the 4–6-month time period							
0–50	35	4	11.4	1	^*^	1	^*^
>50–100	24	7	29.2	3.19	0.82, 12.48	2.12	0.49, 9.20
>100–200	11	6	54.5	9.30	1.92, 45.10	10.44	1.93, 56.45
>200	22	14	63.6	13.56	3.50, 52.63	9.98	2.47, 40.33
Total	92	31	33.7	^*^	^*^	^*^	^*^

^a^Adjusted for age, sex and cancer type.

shows the frequency of skeletal complications and (with the 0–50 group as the normal reference category) both the unadjusted and adjusted odds ratios with 95% confidence intervals for a patient to experience a skeletal complication for each of the four Ntx value groups. There was a strong and highly significant association between baseline Ntx and the occurrence of a skeletal complication (*P*<0.001; test of association). Although the data are not shown, a significant association between baseline Ntx and the occurrence of a skeletal complication was also observed (*P*<0.001) when death was not included as a complication.

Both univariate and multivariate logistic regression models were fitted to the data with any skeletal complication as the dependent variable. With univariate analysis based on the baseline Ntx value, the model correctly predicted 41 (84%) of the 49 patients who would experience a skeletal complication over the 3-month period. In the multivariate analysis, the factors included in the model were age, sex, cancer type and the categorical values of baseline Ntx. Again, the model correctly predicted 41 (84%) of the 49 patients with skeletal complications. The factors age, sex and cancer type did not significantly affect the chances of a patient having a skeletal complication (*P*=0.80, 0.84 and 0.76, respectively), while baseline Ntx was highly significant (*P*<0.001), both in the unadjusted and the adjusted analyses. Patients with a baseline Ntx >100 nmol mmol^−1^ creatinine (i.e. above the normal range) were 19.48 (95% CI 7.55, 50.22) times more likely to experience a complication over the subsequent 3 months than patients with ‘normal’ baseline Ntx values (<100 nmol mmol^−1^ creatinine).

In all, 28 patients had received bisphosphonate therapy prior to monitoring of Ntx levels in this study. However, the associations described above between baseline Ntx and skeletal complications were very similar when these patients were excluded. The adjusted odds ratios for the 93 bisphosphonate naïve patients are shown in Table 5

**Table 5:** Distribution of skeletal complications and adjusted odds ratios according to baseline Ntx range for the 0–3-month period for patients not on bisphosphonate therapy at the time of baseline Ntx measurement

**Baseline Ntx (nmol mmol^−1^ creatinine)**	**Number of patients**	**Number of patients having any skeletal complication**	**% patients having any skeletal complication**	**Odds ratio**^a^	**95% confidence interval**
0–50	23	1	4.3	0.37	0.03, 4.67
>50–100^b^	27	3	11.1	1	^*^
>100–200	20	12	60.0	16.33	2.78, 95.87
>200	23	19	82.6	38.24	6.01, 243.42
Total	93	35	37.6	^*^	^*^

a Adjusted for age, sex and cancer type.

b The >50–100 group was used as the reference category since only one skeletal complication occurred in the Ntx 0–50 nmol mmol^−1^ creatinine range which gave unstable estimates when this range was used as the reference category.

. Again, baseline Ntx was highly significant (*P*<0.001), and the multivariate logistic regression model correctly predicted 28 (80%) of the 35 patients who experienced a skeletal complication during months 0–3. Age, sex and cancer type did not contribute to the multivariate model (all *P*>0.15).

### Analysis of 4–6 month data

Figure 2B shows the scatter plot for the 3-month Ntx value (regarded as a new predictor) against the number of skeletal complications in the subsequent 3-month time period (months 4–6). The Spearman's rank correlation coefficient for this analysis was 0.46 (*P*<0.001). A similar plot for the data using the mean Ntx measurements from months 4, 5 and 6, instead of Ntx at 3 months, also showed a highly significant relationship.

Table 4b shows the frequency of skeletal complications and (again with the 0–50 group as the normal reference category) the unadjusted and adjusted odds ratios with 95% confidence intervals for a patient to experience a skeletal complication during months 4–6 for each of the four Ntx value groups. Once more there was a statistically significant association between the Ntx value at 3 months and the occurrence of skeletal complications during this second 3-month time period (*P*<0.001). As in the initial 3-month time period, patients with a baseline Ntx>100 nmol/mmol creatinine (ie above the normal range) were at significantly higher risk of subsequent complications. The univariate and multivariate logistic regression models correctly predicted 20 (65%) and 18 (58%) of the 31 patients, respectively, who experienced a skeletal complication during months 4–6. The factors age, sex and cancer type were not statistically significant in the multivariate logistic regression model (all *P*>0.3).

### Biochemical response and follow-up for longer than 6 months

Although it was not a primary aim of the study, estimates of response were determined by examining data from patients with a baseline Ntx>50 nmol mmol^−1^ creatinine and who therefore had scope for a biochemical response defined as >50% reduction in Ntx level. Progression was defined as >50% increase and no change as between 50% reduction and 50% increase (Vinholes *et al*, 1997). At 3 months, the data show 15 out of 76 (20%) responders, 36 out of 76 (47%) no change and 25 out of 76 (33%) progression. Response after 3 months, but before 6 months was uncommon, occurring in 4 out of 33 (12%) with 22 out of 33 (67%) showing no change and seven out 33 (21%) progression. The majority of patients on the study received oral clodronate and this response rate is consistent with previous data with this drug. Higher biochemical response rates would be expected with more potent IV bisphosphonates (Vinholes *et al*, 1997; Jagdev *et al*, 2001). A proportion of the patients (approximately 21%) had already been on bisphosphonates prior to study entry and may have already achieved a biochemical response.

For patients monitored for more than 6 months, the numbers of patients for whom results were available decreased progressively from 47 for 7–9 months to three for 22–24 months. There were relatively few skeletal complications and the majority of patients had Ntx values in the normal range, reflecting the previous observation that elevated Ntx is associated with poor prognosis. (Ali *et al*, 2000). Although not appropriate for formal statistical testing, a probable relationship between Ntx and the occurrence of skeletal events remains. Ntx was >100 nmol mmol^−1^ creatinine in eight out 22 (36%) and 11 out of 82 (13%) of the 3-month periods of patient observation with and without an event/death, respectively.

## DISCUSSION

This study in patients with metastatic bone disease has indicated a strong, statistically significant correlation between the rate of bone resorption as measured by Ntx and the frequency of skeletal complications. We are not aware of any previous studies in metastatic bone disease that have related Ntx levels to the range of skeletal complications considered in this study. The baseline Ntx was a statistically reliable predictor of complications over the first 3 months and the 3-month Ntx value was also a strong predictor of complications occurring in the following 3 months. This was true regardless of whether data were limited to patients who were bisphosphonate naive at study entry, and the strength of the correlation is particularly striking in view of the fact that all patients received bisphosphonate therapy after entry to the study.

The multivariate logistic regression model was able to predict correctly 84% of patients who had one or more events in the 0–3-month period, based on baseline Ntx. This is sufficiently accurate to be clinically useful in patient management. For the 4–6-month period, despite the fact that all patients had been on bisphosphonates for at least 3 months, the model was still of predictive value, although less accurate (58% patients correctly identified). The beneficial effects of anticancer treatments and bisphosphonate therapy, as well as the attrition of the worst prognosis patients, probably explain the lower event rate in the second (4–6 months) time period.

Overall, bisphosphonates reduce the frequency of skeletal events by 25–40% (Lipton *et al*, 2000; Berenson *et al*, 2001). However, bisphosphonates are a relatively costly additional intervention in cancer care, which is now applicable to a very large proportion of patients with advanced malignancy. The cost effectiveness has been questioned (Hillner, 2001) and prioritisation of bisphosphonate use is needed. It is not known whether the relative benefits of bisphosphonates are the same across the range of possible bone resorption rates. However, on the assumption that they are, with about one-third of events prevented, our data suggest that the numbers of patients who would need to be treated to prevent one skeletal event would be 31, 17, 3 and 2 for the <50, 50–100, 100–200, and >200 mmol mol^−1^ creatinine levels of Ntx, respectively. A more cost-effective use of bisphosphonates might be to reserve them until patients have Ntx levels above 100 nmol mmol^−1^ creatinine and adjust the dose and schedule to maintain a normal rate of bone resorption.

The current work is consistent with the study by Lipton *et al* (1998), which investigated the fracture rate in 21 cancer patients with bone metastases who received intravenous pamidronate and whose baseline Ntx levels were above the normal range. In the 12 patients who normalised Ntx, five patients (42%) developed fractures, whereas in the nine patients who failed to normalise, eight patients (89%) developed fractures (*P*=0.07). When disease progression was included in the analysis in the above study, the association with Ntx did reach statistical significance (*P*=0.03). Although these data represent only a small number of patients, they also suggested that normalisation of bone resorption should be the goal in reducing fracture rate.

The conclusions of the current study in patients with metastatic bone disease parallel those from studies of bone markers in bone loss due to osteoporosis. In one of the most comprehensive studies, nine bone turnover markers were measured in 375 women aged between 50 and 85 years (Greenfield *et al*, 2000), including Ntx and C-telopeptide (Ctx). This study concluded that high marker levels were associated with an increased risk of vertebral fracture. There are also many osteoporosis studies that have demonstrated the role of bone markers in assessing response to anti-resorptive therapy (Gonnelli *et al*, 1997).

Although urinary Ntx has proved to be one of the most responsive bone markers in metastatic bone disease and results from the present study are encouraging, it would be desirable to study the relationship between skeletal events and other urine and serum bone markers. Serum markers are especially convenient as blood is often being taken for other purposes (though diurnal variation is a problem for serum) and serum measurement avoids complex urine collection and creatinine correction. A recent study of ibandronate in multiple myeloma (Menssen *et al*, 2002) demonstrated that patients experienced significantly fewer skeletal-related events per patient year when the ibandronate dose selected effectively suppressed the bone turnover markers serum osteocalcin and urinary Ctx. This study also found that patients experiencing a defined reduction in both bone markers suffered fewer events than in those with reduction in only one marker, raising the possibility that an index comprising more than one bone marker may add further predictive value.

Large, multicentre trials in metastatic bone disease with the potent bisphosphonate, zoledronic acid have recently been completed. Using the principles and concepts developed in the present study, we are currently analysing the marker and skeletal event data from these trials, to further assess the role of markers in prediction of skeletal complications and determining its value in routine clinical practice.

Further work will also be necessary to demonstrate that patients who normalise their Ntx level following bisphosphonate therapy, experience fewer skeletal events than those who do not normalise. Nevertheless, the clear conclusion of the present work that Ntx levels in the normal range are associated with a much-reduced incidence of skeletal events, together with corresponding previous findings for pain and quality of life, provide strong evidence that normalisation of bone resorption should be a major aim of therapy. This also adds weight to the argument that bone resorption measurements should become a routine part of management of patients with metastatic bone disease.
